# Psychological wellbeing, memories, and future thoughts during the Covid-19 pandemic

**DOI:** 10.1007/s12144-021-01969-0

**Published:** 2021-06-15

**Authors:** Julie A. Niziurski, Marie Luisa Schaper

**Affiliations:** Institute for Experimental Psychology, Heinrich-Heine-Universität Düsseldorf, Düsseldorf, Germany

**Keywords:** Covid-19, Memories, Future thoughts, Wellbeing

## Abstract

The Covid-19 pandemic led countries to place restrictions on the general public in order to protect their safety. These restrictions, however, may have negative psychological consequences as people are restricted in their social and leisure activities and facing daily life stressors. Investigating the relationship between how people are remembering pandemic events and thinking about their futures is important in order to begin to examine the psychological consequences – cognitive and emotional – of the Covid-19 pandemic. The present study examined how characteristics of past and future thinking relate to psychological wellbeing during the Covid-19 pandemic. In an online questionnaire study, 904 participants in Germany and the USA recalled and predicted negative and positive events related to the pandemic. Participants completed a series of questionnaires measuring cognitions and psychological symptoms. Participants’ current psychological wellbeing related to how they remembered events and thought of their future. Participants reported a greater sense of reliving for past compared to future events. However, future events were more rehearsed than past events. Additionally, the emotional impact of positive and negative events differed for the past and the future. Participants seem to be strongly future oriented during the Covid-19 pandemic, but have a negative view of future events.

The initial wave of the Covid-19 pandemic was a global phenomenon that affected almost everyone in some way. There were large-scale lockdowns in many countries, restrictions on travel, social distancing, and unexpected financial and social stressors. The worldwide restrictions and safety guidelines were meant to protect the physical health of the world’s citizens. However, some early research on the Covid-19 pandemic demonstrated the negative consequences these restrictions had on mental health (Tull et al., 2020; see Brooks et al., 2020 for review). Memories and future thoughts are related to wellbeing (Conway & Pleydell-Pearce, 2000). Therefore, how people are remembering events and imaging their future during the Covid-19 pandemic, should relate to their psychological wellbeing. In the present study, we examined how individuals remembered negative and positive events that occurred during the Covid-19 lockdowns and how, in light of the pandemic, they were thinking about positive and negative future events. We examined how the characteristics of individuals’ memories and future thoughts (e.g., emotional impact, sense of reliving, and rehearsal) were related to current levels of psychological symptoms.

Autobiographical memories are memories for our personal past (Rubin, 1986). Such memories influence how we think of our future. In fact, it is theorized that during evolution, autobiographical memory developed to allow us to envision the future (Schacter & Addis, 2007; Schacter et al., 2007). The connection between memories and future thoughts is further corroborated by studies which show both memories and future thoughts recruit the same neural networks and respond similarly to experimental manipulations (see Finnbogadóttir & Berntsen, 2011, for review). One notable difference between memories and future thoughts is that memories are more vivid (e.g., have a greater sense of reliving) than future thoughts (Cole & Berntsen, 2016). Thoughts about our futures, which are of events that have not yet occurred, require more effort to produce and are largely schema-driven, thus have a weaker sense of living than memories (see Berntsen & Bohn, 2010 for review).

## Psychological Symptoms and Emotional Cognitions

Past and future cognitions have repeatedly been shown to be associated with psychological wellbeing. For one, stress has been shown to relate to, or even predict, poor psychological wellbeing (see Cox, 1978, 1987 for reviews). Both the hopelessness theory (Abrasmson et al., 1989) and Beck’s cognitive theory of depression (Beck, 1987) hypothesize that cognitive styles and attitudes interact with stress to elevate other psychological symptoms.

Anxiety is related to both past and future thinking. For one, anxiety is strongly future oriented (Beck et al., 1987) and anxious individuals find it easier than non-anxious controls to think of negative future events and also believe those negative future events will occur (MacLeod & Byrne, 1996; MacLeod et al., 1996; also see Wu et al., 2015). Additionally, anxious individuals have a memory bias towards recalling anxiety-related memories and have self-defining memories related to their anxiety (Krans et al., 2013; Sutherland & Bryant, 2008).

Depression and anxiety are often highly correlated (Hankin et al., 2004). Individuals with depression show a *negativity bias*, that is, have a negative view of themselves, the world, and their futures (Beck, 1967, 1987). Individuals with depression find it easier to recall negative than positive information (see Dalgleish & Werner-Seidler, 2014 for review). For example, when given a positive retrieval prompt (e.g. birthday), individuals with depression will struggle to recall specific events related to the prompt. Instead they will retrieve an overgeneral memory (e.g., I have chocolate cake on my birthday) and they find difficult to give more details (Williams et al., 1996; Williams et al., 2007). Additionally, as memories influence thoughts of the future (Schacter & Addis, 2007; Schacter et al., 2007), individuals with depression have a negative outlook on their future (Beck, 1987; also see hopelessness theory: Abramson et al., 1989). In contrast, positive future thinking is related to lower psychological symptoms, such as hopelessness (O'Connor et al., 2004). Negative thinking is also seen as a risk factor for developing psychological disorders, such as Depression (see Watters & Williams, 2011 for review). Thus, psychological symptoms and cognitions seem to have a reciprocal relationship, in that positive and negative cognitions affect psychological wellbeing and distress and vice versa.

## Present Study

In the present study, we examined how individuals remembered negative and positive events that occurred during the Covid-19 lockdowns and how, in light of the pandemic, they were thinking about positive and negative future events. We examined how the characteristics of individuals’ memories and future thoughts (e.g., emotional impact, sense of reliving, and rehearsal) were related to their levels of depression, anxiety, and stress. We recruited participants in Germany and the USA. These are countries that have, in general, similar qualities (e.g., Western, Industrialized, rich etc.). Both countries experienced largescale lockdowns at the time of data collection, which was during the early stages of the pandemic (April/May 2020). If we find a normal range of psychological symptoms (i.e., normal range on the DASS-21), negative and positive memories should have similar emotional impact and be rehearsed equally often as negative and positive future thoughts (Schacter et al., 2007). However, memories should have a higher sense of reliving than future thoughts (Berntsen & Bohn, 2010).

Due to the negativity bias in individuals with depression, as depressive symptoms increased, negative memories should be rated as more emotionally intense and more often rehearsed, and vice versa for positive memories (Beck, 1967). However, as depressive symptoms increased, memories should be overgeneralized, and, thus, rated to have a lower sense of reliving (Williams et al., 1996). As anxiety and stress levels increased, negative and positive memories should be rated as highly emotional, have a higher sense of reliving, and be often rehearsed (Beck, 1987; Walker et al., 2014).

Further, supported by the negativity bias in depression, as depression levels increased, individuals should have a more negative view of their future (Abramson et al., 1978; Beck, 1967). This would be evident in higher emotional impact of negative future events and lower emotional impact of positive future events. As anxiety is future oriented, as anxiety levels increased, negative future events should have higher ratings of emotional impact, sense of reliving, and rehearsal (Beck et al., 1987). A similar pattern should be found in those with high levels of stress (cf. Beck, 1987).

## Method

### Participants

The Ethics Committee of the Heinrich-Heine-University Düsseldorf approved the study. Participants were recruited via social networks in Germany and the USA for an online study. Data was collected over a two-week period from April 20th to May 4th (CEST). During this time, both Germany and the USA were experiencing large scale lockdowns and increases in Covid-19 cases (Johns Hopkins University, 2020). We recruited 905 participants. One participant responded the same for all question and thus was removed. A sensitivity analysis using G*Power (Faul et al., 2007) showed that with *N* = 904 correlation effects of ρ = .12 (a small effect, cf. Cohen, 1988) could be detected with power 1 – β = .95 and α = .05 (two-tailed). Participants’ age ranged from 18 to 78 (*M* = 32.09, *SE* = 0.46, 35 non-responder). We had 658 females, 228 males, 7 non-binary, and 11 non-responders. We also assessed nationality, country of residence, education, household size, and communication with others (see Table 1 for frequencies).

**Table 1:** Nationality, Education Level, and Additional Demographic Information

Nationality						
Unites States of America	Germany	Other				No response
210 (23.3%)	646 (71.6%)	30 (3.3%): Israeli (5), British (3), French (2), Brazilian (2), Luxembourgish (2), Austrian (2), Greek (2), Belgian, Canadian, Chinese, Indian, Italian, Polish, Russian, Spanish, Swiss, Syrian, Thai, and Ukrainian (1 each).				18 (2.0%)
Country of Residence						
United States of America 223			Germany 681			
Education						
Graduate Degree	Undergraduate Degree	Trade School	High School Diploma		Secondary School	Other
241 (26.7%)	246 (27.2%)	27 (3.0%)	329 (36.4%)		44 (4.9%)	17 (1.9%)
Household Size						
0	1	2	3	4	5+	No response
144 (16.1%)	296 (33.1%)	214 (23.9%)	137 (15.3%)	71 (7.9%)	33 (3.7%)	9 (1%)
Contact with others						
Meet in Person	Social Media	Email	Video Chat	Phone Calls	Letters	No Contact with Others
401 (44.4%)	763 (84.4%)	332 (36.7%)	711 (78.7%)	753 (83.3%)	109 (12.1%)	22 (2.4%)

*Note.* Household Size is the number of people in one’s household other than one’s self. Household Size: Range = 0–15; *M* = 1.81; *SE* = .05. Participants could choose more than one option for “Contact with Others”

### Materials, Procedure and Design

Participants first provided informed consent. The present study was part of a larger questionnaire study, but here we focused on the DASS-21 and the Autobiographical Memory Questionnaire:

1. DASS-21 (Lovibond & Lovibond, 1995; German version: Nilges & Essau, 2015) is a 21 item questionnaire measuring symptoms of depression, anxiety, and stress over the past week. There are seven questions for each of the three subscales: depression, anxiety, and stress. Each question is answered on a 4-point Likert scale from 0 (*did not apply to me at all - NEVER*) to 3 (*applied to me very much, or most of the time - ALMOST ALWAYS*). The subscale scores are calculated individually by adding up the values of participants’ responses to the seven questions for one subscale and multiplying it by 2 (cf. Lovibond & Lovibond, 1995). The traditional cutoff scores were used to determine the range participants’ scores fell in (e.g., normal, mild, etc.; Lovibond & Lovibond, 1995).
2. Autobiographical Memory Questionnaire (AMQ; Rubin et al., 2003) is a 17 item questionnaire measuring retrieval characteristics and emotional reaction to memories. We adapted the AMQ to examine memories and future thoughts (e.g., Rubin, 2014). The questionnaire was translated and back-translated from English to German by two independent researchers (English version available in the Online Supplement; Niziurski & Schaper, 2020). The AMQ has three subscales: 1. Emotion, measuring emotionality and emotional impact of the thought, 2. Reliving, the sense of (re-)experiencing the thought and sensory details, and 3. Rehearsal, how often the person thinks of the thought, voluntarily or involuntarily (for examples, see Supplement Material). Questions were answered on a 7-point Likert scale (1 = Not at all/Almost Never; 7 = To a Great Extent/Very Often). Five additional questions regarding the foreseeability and inevitability of the events were not pertinent to the present study.

Participants completed four AMQs on events associated with the Covid-19 pandemic: 1. past negative event, 2. future negative event, 3. past positive event, 4. future positive event. Participants completed one round of the AMQ for each thought. At the start of the AMQ, participants were instructed which thought they should be thinking about. For example, the instructions for the past positive event read: “Please think of the most positive event that has happened in your life because of the COVID-19 pandemic.” Each of the four times the participants completed the AMQ, they first described the recalled memory/imagined future thought and then answered the rating questions for the subscales. For ethical reasons, participants: 1. were allowed to leave the description blank, but were instructed to think about the memory/future thought as they responded and 2. answered the AMQ in the same order (ending with positive thoughts) to limit negative side effects of participation. Participants were asked to continue to think of their memory/future event even if they did not provide a description: “If you do not want to give a description, you do not have to. You may leave this question blank. However, please continue to think of the event as you answer the following multiple choice questions.”

Lastly, participants completed a demographic questionnaire and could sign up to be contacted for follow-up studies. On average participants completed the questionnaires in 35–40 min. For each participant who completed the questionnaire, we donated 3€ (German participants) or $3 (US participants) to the Red Cross. German students could instead choose to receive course credit.

## Results

The data and supplementary analyses are available at https://osf.io/6zq83/ (Niziurski & Schaper, 2020).

### Autobiographical Thoughts

Autobiographical thoughts were organized by valence (positive/negative) and time (past/future) based on the instructions participants received during the respective AMQ (e.g., positive past: “Please think of the most positive event that has happened in your life because of the COVID-19 pandemic.”). We aimed to further elucidate the contents and themes of these thoughts. For both positive and negative thoughts, two raters generated ten categories each (see Table 2 for categories and frequencies^1^). Separate categories were generated for the negative and positive thoughts because positive thoughts (past and future) shared similar themes and the same was true for the negative thoughts. Once the categories were created, memories and future thoughts were then coded by two new independent judges with an initial interrater agreement of 88%. Coding disagreements were solved by a third independent judge and the final interrater agreement was 100%.

**Table 2:** Coding of Memories and Future Thoughts

		Time	
Valence	Topic	Past Memories	Future Thoughts
Negative	Conflict in Relationships	50	7
	Sickness/Death of Family/Friend/Self	111	237
	Cancelled Activities	24	7
	Cancelled Plans/Events	63	34
	Other Daily Life Hassles	24	6
	Economic Loss/Job Loss	65	105
	Academic/Education Hardships	51	61
	Social Isolation/Loneliness (self or others)	162	37
	Other	20	21
	No Negative Event	9	2
	Total (%)	579 (64.05%)	517 (57.19%)
Positive	Improved Relationships	187	69
	Economic/Job Improvement	36	61
	Improved Work/Life Balance	112	53
	Academic/Education Improvements	24	40
	Positive Changes for Environment	3	10
	Positive Changes for Society	40	43
	Cure/Vaccine	2	11
	Able to Attend Events/Travel	4	22
	Other	24	20
	No Positive Event	6	17
	Total (%)	438 (48.45%)	346 (38.27%)

*Note*. For ethical reasons, participants were not required to give a description. Therefore, the total number of coded memories is dependent on how many participants gave a description for a particular valence-time combination (e.g., positive-future). % is the percent of participants who gave descriptions out of the 904 participants

### Depression, Anxiety, and Stress

**Fig. 1:**
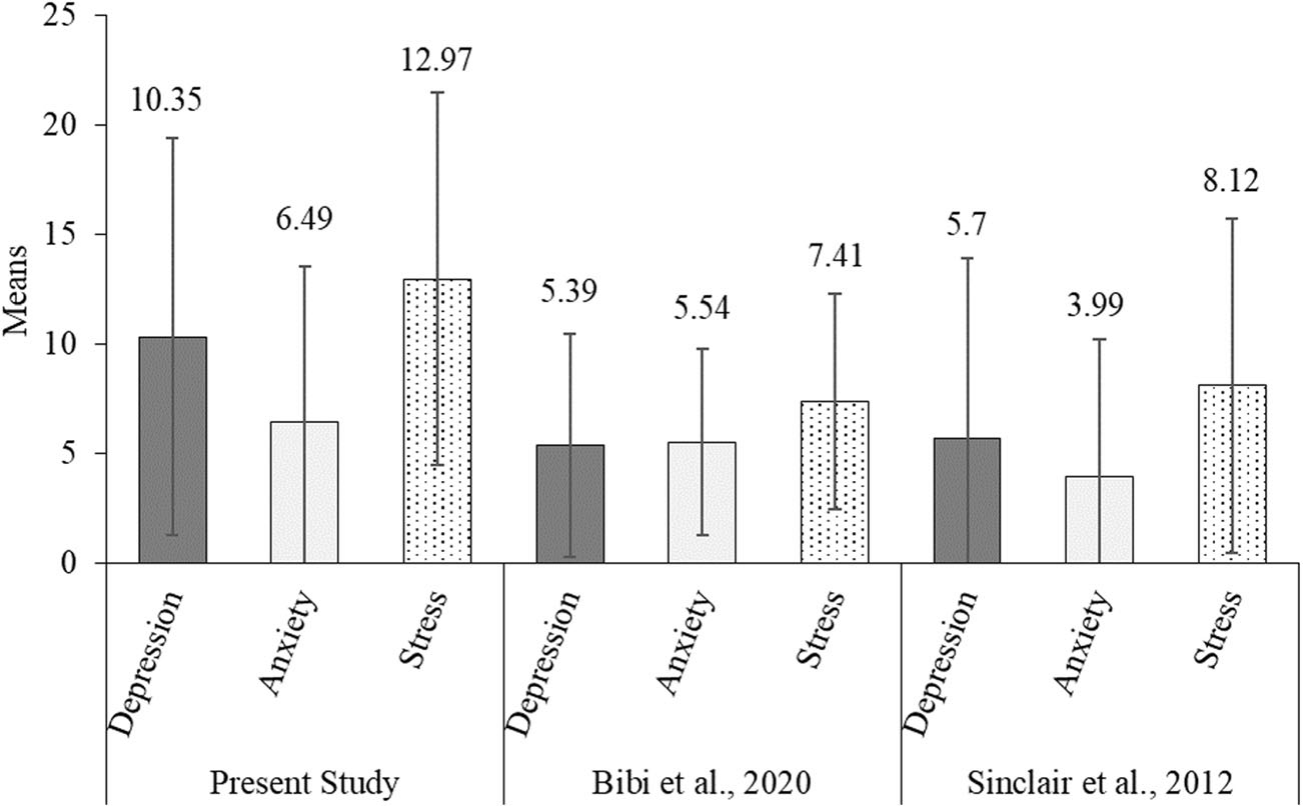
Comparison of Present study’s DASS-21 mean scores to those of similar studies conducted prior to the Covid-19 pandemic. The numbers above each bar is the Mean. For the present study, the Depression scores ranged from 0 to 42, Anxiety scores ranged from 0 to 38, and Stress scores ranged from 0 to 42. Error bars indicate standard deviations

Depression, anxiety, and stress were on average in the normal to mild range on the DASS-21 scales (see Fig. 1 for means).^2^ We deem it important to note that the means for the present study were elevated compared to recent studies conducted with samples similar to ours in Germany (Bibi et al., 2020) and the USA (Sinclair et al., 2012) prior to the pandemic (see Fig. 1). Note that these are not randomized control groups, and as such, a causal interpretation of any differences needs to be taken with caution. We compared the DASS-21 subscale values obtained from the present sample with the subscale values of the two previous studies by Bibi et al. (2020) and Sinclair et al. (2012) using between-subjects *t* tests (based on the statistics reported in these papers). All comparisons were significant, all *p*s < .001. This was also the case if we compared the current German sample with the German sample of Bibi et al. and the present US sample was compared to the US sample of Sinclair et al., all *p*s < .041. Thus, our sample reported significantly elevated levels of depression, anxiety, and stress compared to samples prior to the pandemic.

### Psychological Distress and Cognitions

**Fig. 2:**
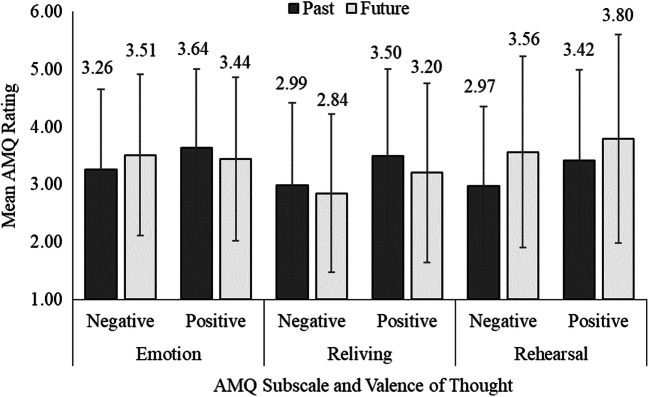
Displayed are the means for the AMQ subscales emotion, reliving, and rehearsal split by valence of the thought (positive/negative) and time (past/future). The rating scales go from 1 to 7. Error bards indicate standard deviations

Figure 2 displays descriptive statistics for the AMQ subscales (emotion, reliving, rehearsal) split by valence of the thought and time. To investigate the relationships between psychological distress and pandemic-related cognitions, we ran linear mixed regression models (e.g., Krull & Mackinnon, 2001) on the three subscales of the AMQ (emotion, reliving, rehearsal; see Online Supplement for the full correlation Table S1). We used the R packages lme4 and lmerTest (Bates et al., 2015; Kuznetsova et al., 2017; R Core Team, 2020), with participants as random effects.^3^ We will now present the results for each subscale.

#### Emotion

We calculated models with the AMQ emotion subscale values as criterion and time (0 = past, 1 = future, centered to participant mean), valence (0 = negative, 1 = positive, centered to participant mean), stress, anxiety, and depression (all centered to grand mean, rescaled by dividing by 10) and all interactions as predictors (cf. Enders & Tofighi, 2007, for justification of centering of predictors). The results for this full model are in Table 3 (first column, see Online Supplement Table S2 for complete inference statistics). We will present the significant results organized by predictor.^4^

**Table 3:** Effects of Stress, Anxiety, Depression, Valence and Time on the AMQ Emotion Scale

		Valence	
Predictor	Full model	Negative	Positive
Intercept	3.54*	3.46*	3.61*
Valence	0.16*		
Stress × Valence	−0.17*		
Stress	0.27*	0.36*	0.19*
Anxiety × Valence	−0.05		
Anxiety	0.33*	0.35*	0.30*
Depression × Valence	−0.43*		
Depression	0.03	0.24*	−0.19*
Time × Valence	−0.41*		
Time	0.02	0.23*	−0.18*
Stress × Anxiety × Valence	−0.21*		
Stress × Anxiety	−0.13	−0.03	−0.24*
Stress × Depression × Valence	0.04		
Stress × Depression	−0.01	−0.03	0.01
Anxiety × Depression × Valence	0.19*		
Anxiety × Depression	−0.08	−0.17	0.02
Stress × Time × Valence	−0.11		
Stress × Time	−0.03	0.02	−0.08
Anxiety × Time × Valence	0.10		
Anxiety × Time	0.05	0.00	0.10
Depression × Time × Valence	0.09		
Depression × Time	0.08	0.03	0.13
Stress × Anxiety × Depression × Valence	−0.02		
Stress × Anxiety × Depression	0.04	0.05	0.03
Stress × Anxiety × Time × Valence	−0.19		
Stress × Anxiety × Time	0.03	0.13	−0.07
Stress × Depression × Time × Valence	0.06		
Stress × Depression × Time	0.03	0.00	0.07
Anxiety × Depression × Time × Valence	0.04		
Anxiety × Depression × Time	−0.04	−0.06	−0.02
Stress × Anxiety × Depression × Time × Valence	0.09		
Stress × Anxiety × Depression × Time	−0.04	−0.04	−0.04

*Note.* Estimates are unstandardized regression weights. Stress, Anxiety, and Depression measures with the DASS-21 (Lovibond & Lovibond, 1995) were centered to the grand mean and rescaled by dividing by 10. Time (0 = past, 1 = future) and Valence (0 = negative, 1 = positive) were centered to the participant mean

* *p* < .05

##### Valence

For our purposes, two results are especially noteworthy from the full model. First, valence of the memory was associated with the emotion subscale. That is, positive events were rated to have a higher emotional impact than negative events (see also Fig. 2). Second, valence showed two-way interactions with stress, depression, and time, and three-way interactions with stress and anxiety, and anxiety and depression. These interactions indicated that these effects differed for negative and positive events. We therefore calculated two follow-up models separated by valence (i.e., for positive and negative events). These results are also in Table 3 (second and third column, see Online Supplement Table S3 for complete inference statistics).

##### Stress

The significant stress × valence interaction in the full model shows that the effect of stress differed between negative than positive events. From the follow-up models, one can see that higher stress symptoms were associated with greater emotional impact of both memories and future thoughts as indicated by the positive regression weights, and this effect was stronger for negative than positive events.

##### Anxiety

As there was no significant anxiety × valence interaction in the full model, the effect of anxiety did not differ between positive and negative events. In all models, higher anxiety symptoms were associated with higher emotional impact for both positive and negative events as indicated by the positive regression weights.

Further, there was a significant three-way stress × anxiety × valence interaction in the full model. From the follow-up models, one can see that for positive events, but not for negative events, there was a significant stress × anxiety interaction. This negative regression weight of this interaction indicated that the effect of stress (i.e., more stress related to higher emotional impact) was weaker in participants with higher levels of anxiety (and vice versa) for positive events only.

##### Depression

The significant depression × valence interaction in the full model shows that the effect of depression differed between negative than positive events. From the follow-up models, one can see that for negative events, higher depression symptoms were associated with higher emotional impact, whereas for positive events, higher depression symptoms were associated with weaker emotional impact. There was further a three-way depression × anxiety × valence interaction in the full model, however, in the follow-up models, neither of the regression weights was significant. Thus, we refrain from interpretation of this effect.

##### Time

The significant time × valence interaction in the full model shows that the effect of time differed between negative and positive events. From the follow-up models, one can see that for negative events, future thoughts were associated with higher emotional impact than memories as indicated by the positive regression weight. For positive events, memories were associated with higher emotional impact than future thoughts, as indicated by the negative regression weight. These effects are also displayed in Fig. 2. All other effects were non-significant.

#### Reliving

We calculated models with the AMQ reliving subscale values as criterion. The predictor setup was the same as for the emotion subscale. The results for this full model are in Table 4 (first column, see Online Supplement Table S4 for complete inference statistics). We will present the significant results organized by predictor.

**Table 4:** Effects of Stress, Anxiety, Depression, Valence and Time on the AMQ Reliving Scale

		Valence	
Predictor	Full model	Negative	Positive
Intercept	3.22*	2.99*	3.45*
Valence	0.46*		
Stress × Valence	−0.10		
Stress	0.22*	0.27*	0.16
Anxiety × Valence	−0.09		
Anxiety	0.31*	0.35*	0.26*
Depression × Valence	−0.26*		
Depression	−0.02	0.10	−0.15*
Time × Valence	−0.17		
Time	−0.21*	−0.13*	−0.30*
Stress × Anxiety × Valence	−0.19		
Stress × Anxiety	−0.11	−0.02	−0.20
Stress × Depression × Valence	0.02		
Stress × Depression	−0.06	−0.07	−0.04
Anxiety × Depression × Valence	0.09		
Anxiety × Depression	−0.08	−0.12	−0.05
Stress × Time × Valence	0.06		
Stress × Time	−0.07	−0.11	−0.03
Anxiety × Time × Valence	0.00		
Anxiety × Time	0.02	0.03	0.02
Depression × Time × Valence	−0.12		
Depression × Time	0.03	0.09	−0.03
Stress × Anxiety × Depression × Valence	0.01		
Stress × Anxiety × Depression	0.06	0.06	0.06
Stress × Anxiety × Time × Valence	0.00		
Stress × Anxiety × Time	0.01	0.01	0.01
Stress × Depression × Time × Valence	−0.04		
Stress × Depression × Time	0.01	0.03	−0.01
Anxiety × Depression × Time × Valence	0.08		
Anxiety × Depression × Time	−0.05	−0.08	−0.01
Stress × Anxiety × Depression × Time × Valence	0.03		
Stress × Anxiety × Depression × Time	0.01	0.00	0.02

*Note.* Estimates are unstandardized regression weights. Stress, Anxiety, and Depression measures with the DASS-21 (Lovibond & Lovibond, 1995) were centered to the grand mean and rescaled by dividing by 10. Time (0 = past, 1 = future) and Valence (0 = negative, 1 = positive) were centered to the participant mean

* *p* < .05

##### Valence

As with the emotion subscale, valence of the memory was associated with the reliving subscale. That is, positive events were rated to have been relived more than negative events (see also Fig. 2). Further, valence showed a two-way interaction with depression. This interaction indicated that the effect of depression differed for negative and positive events. We therefore again calculated two follow-up models separated by valence. These results are also in Table 4 (second and third column, see Online Supplement Table S5 for complete inference statistics).

##### Stress

As there was no significant stress × valence interaction in the full model, the effect of stress did not differ between positive and negative events. In the full model, higher stress symptoms were associated with a stronger sense of living as indicated by the positive regression weight. This effect was also significant in the follow-up model for negative events, but not for positive events (however, there was no significant stress × valence interaction in the full model).

##### Anxiety

As there was no significant anxiety × valence interaction in the full model, the effect of anxiety did not differ between positive and negative events. In all models, higher anxiety symptoms were associated with a stronger sense of reliving as indicated by the positive regression weights.

##### Depression

The significant depression × valence interaction in the full model shows that the effect of depression differed between negative than positive events. As one can see from the follow-up models, higher depression symptoms were associated with a weaker sense of reliving for positive events only as indicated by the negative regression weight. For negative events, there was no such effect.

##### Time

As there was no significant time × valence interaction in the full model, the effect of time did not differ between positive and negative events. In all models, past events were more strongly relived than future events as indicated by the negative regression weights. This can also be seen from Fig. 2. All other effects were non-significant.

#### Rehearsal

We calculated models with the AMQ rehearsal subscale values as criterion. The predictor setup was the same as for the emotion and reliving subscales. The results for this full model are in Table 5 (first column, see Online Supplement Table S6 for complete inference statistics). We will present the significant results organized by predictor.

**Table 5:** Effects of Stress, Anxiety, Depression, Valence and Time on the AMQ Rehearsal Scale

		Valence	
Predictor	Full model	Negative	Positive
Intercept	3.54*	3.35*	3.74*
Valence	0.40*		
Stress × Valence	−0.06		
Stress	0.31*	0.34*	0.28*
Anxiety × Valence	0.03		
Anxiety	0.26*	0.24*	0.28*
Depression × Valence	−0.29*		
Depression	0.00	0.14*	−0.15
Time × Valence	−0.24*		
Time	0.51*	0.63*	0.39*
Stress × Anxiety × Valence	−0.10		
Stress × Anxiety	−0.11	−0.06	−0.16
Stress × Depression × Valence	−0.02		
Stress × Depression	−0.11	−0.10	−0.11
Anxiety × Depression × Valence	−0.04		
Anxiety × Depression	−0.07	−0.05	−0.09
Stress × Time × Valence	0.04		
Stress × Time	−0.08	−0.10	−0.06
Anxiety × Time × Valence	−0.02		
Anxiety × Time	0.03	0.04	0.02
Depression × Time × Valence	−0.03		
Depression × Time	0.12	0.13	0.10
Stress × Anxiety × Depression × Valence	0.03		
Stress × Anxiety × Depression	0.07	0.05	0.08
Stress × Anxiety × Time × Valence	0.20		
Stress × Anxiety × Time	0.09	−0.01	0.19
Stress × Depression × Time × Valence	0.02		
Stress × Depression × Time	−0.05	−0.06	−0.04
Anxiety × Depression × Time × Valence	−0.22		
Anxiety × Depression × Time	−0.09	0.01	−0.20
Stress × Anxiety × Depression × Time × Valence	0.01		
Stress × Anxiety × Depression × Time	0.01	0.01	0.02

*Note.* Estimates are unstandardized regression weights. Stress, Anxiety, and Depression measures with the DASS-21 (Lovibond & Lovibond, 1995) were centered to the grand mean and rescaled by dividing by 10. Time (0 = past, 1 = future) and Valence (0 = negative, 1 = positive) were centered to the participant mean

* *p* < .05

##### Valence

As with the emotion and reliving subscales, valence of the memory was associated with the rehearsal subscale. That is, positive events were rated to have been rehearsed more than negative events (see also Fig. 2). Further, valence showed two-way interactions with depression and time. These interactions indicated that these effect differed for negative and positive events. We therefore calculated two follow-up models separated by valence. These results are also in Table 5 (second and third column, see Online Supplement Table S7 for complete inference statistics).

##### Stress

As there was no significant stress × valence interaction in the full model, the effect of stress did not differ between positive and negative events. In all models, higher symptoms of stress were associated with generally more rehearsal of events as indicated by the positive regression weights.

##### Anxiety

As there was no significant anxiety × valence interaction in the full model, the effect of anxiety did not differ between positive and negative events. In all models, higher symptoms of anxiety were associated with generally more rehearsal of events as indicated by the positive regression weights.

##### Depression

The significant depression × valence interaction in the full model shows that the effect of depression differed between negative than positive events. As can be seen from the follow-up models, higher depression symptoms were associated with more rehearsal of negative events only as indicated by the positive regression weight. There was no such effect for positive events.

##### Time

The significant time × valence interaction in the full model shows that the effect of time differed between negative than positive events. In all models, future events were rehearsed more than past events as indicated by the positive regression weights. This effect was stronger for negative than for positive events. This can also be seen from Fig. 2. All other effects were non-significant.

## Discussion

The Covid-19 pandemic is a global event which has presented individuals with uncontrollable situations. Current psychological wellbeing influences how people remember and predict events. How memories are recalled and future thoughts envisioned can also influence psychological symptoms and thus a vicious circle is created. Here, individuals in Germany and the USA rated characteristics (emotional impact, sense of reliving, rehearsal) of memories and future thoughts related to the pandemic. We examined how their current psychological wellbeing (depression, anxiety, and stress) related to these thoughts. Depression, stress, and anxiety showed differential relationships to the emotional impact, sense of reliving, and rehearsal of past and future negative and positive events.

### Autobiographical Memories Vs. Future Thoughts

In general, positive events in the past and future were rated to have more emotional impact, greater sense of reliving, and were more often rehearsed compared to negative events. This constitutes a positivity bias in autobiographical thoughts. In previous research such a positivity bias was strongest in future thinking, but still present in past thinking (see Walker et al., 2003, for review). This general positivity bias may be adaptive in keeping a positive image of one’s self (Rasmussen & Berntsen, 2013). Positive future thinking serves an adaptive function as it allows people to imagine an idyllic future. This in turn may allow them to seek new experiences, despite potential risk for failure or disappointment (Taylor & Brown, 1988).

Although we collected data in the unique situation of the Covid-19 pandemic, we were able to replicate previous findings showing that memories were rated with a higher sense of reliving compared to future events (Berntsen & Bohn, 2010; Rasmussen & Berntsen, 2013). However, emotional impact differed depending on the valence of the event. Negative future events were rated as more emotionally impactful than positive future events, whereas positive memories were rated as more emotionally impactful than negative memories. The differences in the effects on emotional impact for memories can be explained by the fading affect bias (Walker et al., 2003): In healthy individuals, the intensity of negative memories fades faster than that of positive memories, lessening the emotional impact of negative memories. As the pandemic began affecting the world in early 2020 and data collection took place in April 2020, some time may have passed from when the participant-elected negative and positive events occurred and thus there was time for the intensity to fade for negative memories. This suggests that fading affect bias is adaptive during this pandemic. In contrast, individuals have the general tendency to think of their futures in a positive light. Thus, when cued to think of a negative future thought, participants in the present study may have been forced to think of an event in which they imagined failure or disappointment. These future negative events were rated as more emotionally intense as they may have been more likely to go against one’s sense of self and negatively impact the mood of the participants (Conway, 2005; Rasmussen & Berntsen, 2013; Taylor & Brown, 1988).

Interestingly, future thoughts were more rehearsed than memories with a stronger effect for negative than positive thoughts. By contrast, previous research has not found a difference in the rehearsal of memories and future thoughts (Cole & Berntsen, 2016) or positive future events to be more rehearsed than negative future events (Özbek et al., 2017). This suggests that individuals may be more focused on potential negative future events during the pandemic. Thinking of negative future events is not always maladaptive (Rasmussen & Berntsen, 2013). It may allow the individual to prepare for a negative outcome or adjust behavior or cognition to avoid potential negative events (proactive coping: Aspinwall & Taylor, 1997; also see Aspinwall, 2011).

### Autobiographical Thoughts and Psychological Symptoms

In the current study, depressive symptoms, anxiety, and stress were elevated compared to pre-pandemic studies (Bibi et al., 2020; Sinclair et al., 2012). This may suggest generally elevated levels of psychological distress in the general population during the Covid-19 pandemic. Increased daily stressors and the unpredictability of the pandemic could relate to worsening psychological wellbeing. While all psychological symptoms were correlated in the present study (see Table S1 in Online supplement), each individually explained parts of the variance for each subscale.

#### Depression

Depressive symptoms related to all characteristics for negative and positive events. Negative events were rated with higher emotional impact as depressive symptoms increased, whereas positive events were rated with lower emotional impact as depressive symptoms increased. Additionally, depressive symptoms were related to increased rehearsal of negative thoughts. These findings demonstrate a negativity bias in those with increased depressive symptoms, which is often found in both memories and future thinking (Abramson et al., 1989; Beck, 1987). Those with higher depressive symptoms also found it difficult to recall or imagine sensory details of positive events (lower ratings of reliving) which could be explained by the tendency for individuals with depression to produce overgeneral memories (Williams et al., 1996; Williams et al., 2007).

#### Anxiety

Higher anxiety symptoms were associated with higher ratings of emotional impact, reliving, and rehearsal for both negative and positive events (Beck et al., 1987; Conway, 2005). These results are consistent with previous findings suggesting that anxiety is related to an amplification of negative and positive emotions (Skowronski et al., 2014; Walker et al., 2014). The emotion dysregulation model suggests this is a result of an inability to properly regulate one’s emotions (Mennin et al., 2004) and is consistent with findings in clinical and non-clinical samples of anxiety disorders, such as PTSD (see Niziurski et al., 2017, for review).

#### Stress

While an increase in stress was related to higher ratings of emotional impact for negative and positive events, the relationship was stronger for negative events. Additionally, increases in stress related to the sense of reliving for negative events only, whereas rehearsal was increased for both negative and positive events. These findings are in line with past research which demonstrates the negative impact of stress on psychological wellbeing (see Cox, 1978, 1987 for reviews). Stress is certainly elevated during the Covid-19 pandemic. Persistent stress can lead to other psychological symptoms (e.g., depression or anxiety). Therefore, future research should continue to monitor stress levels in the general population in connection to developments of the pandemic and how increases in stress may relate to the development of clinical disorders.

### Limitations & Future Directions

The present study collected data from two rather similar opportunity samples in Germany and the USA. Our current results are therefore limited to these two countries. As the Covid-19 pandemic is an international event, future studies should include more countries in their samples, for example, comparisons between Western and Eastern societies.

One might have expected differences in psychological distress between the USA and Germany. Kessler and Broment (2013) reported higher levels of depression in the USA than Germany. Further, the reactions of the political leadership in the early stages of the pandemic were quite different, with Germany responding overall faster than the USA (Farr, 2020). This might have influenced participants psychological distress and cognitions about the pandemic. That said, our current results were, in this regard, rather inconclusive. Participants from the USA reported higher levels of stress and anxiety (see Footnote 2), but not depression as reviewed by Kessler and Bromet (2013). Participants’ cognitions’ involved the same themes (see Table 2), irrelevant of country of residence. Further, differences in stress and anxiety did not systematically change the relationships between the DASS-21 scales and the AMQ scales (see Footnote 4). Further research is therefore needed to test for systematic differences in psychological distress and pandemic-related conditions across cultures around the world.

Additionally, the design of the experiment allowed participants to answer the AMQs without providing a written description of the thought they were prompted to think of (e.g., a positive memory related to the pandemic). As explained in the Method section, this was done for ethical reasons and has been done in other studies (e.g., Niziurski et al., 2018; Niziurski & Berntsen, 2018). We are unable to determine why people chose to provide descriptions for some prompts and not others. We hypothesize one reason could be fatigue, as the AMQs occurred toward the end of the experiment (see Footnote 1). Another possibility is that people found it difficult to respond to some prompts compared to others (e.g., easier to think of a negative future event compared to a positive one). However, if this were the case, then we would see lower ratings on all AMQ subscales as less clear thoughts would have less emotional impact, a lower sense of reliving, and be less rehearsed. Therefore, a description is not necessary to measure how a thought impacts the individual as this can be seen in the ratings. Future research could emphasize the necessity to provide a description given that appropriate ethical procedures are used to ensure participants’ wellbeing.

### Broader Perspective

The results of the present study largely replicate what is found in the memory and mental health literatures (see Watson & Berntsen, 2015 for review). We think that this is promising news for psychologists and mental health professionals across the world. The Covid-9 pandemic was, and still is, a unique global situation and thus, it was and is difficult to predict how people’s metal health would react. The present study demonstrates that whereas psychological symptoms were elevated and participants thought more negatively of their futures than their pasts (opposite of what was found prior to the pandemic), the typical relationships between cognition and mental health replicated (e.g., negativity bias in those with depressive symptoms; elevated ratings on all AMQ subscales for all thoughts – negative and positive -- in those with high anxiety symptoms; and greater impact of negative thoughts in those with high levels of stress). Thus, as the general patterns of thinking seem to be the same as prior to the pandemic, traditional evidence-based cognitive therapies, such as cognitive behavior therapy (see Hofmann et al., 2012 for review)), should very likely still be useful for dealing with psychological symptoms related to the pandemic. Thus, the present study suggests that whereas more people may be seeking psychological help due to the pandemic, traditional cognitive therapies can be used as the traditional relationships between cognitions and mental health are still prevalent, even during the pandemic. Of course, longitudinal studies are needed to ensure that such interventions improve mental health issues associated with the pandemic.

### Conclusion

The Covid-19 pandemic is a unique global event. As the virus itself is currently unpredictable, so are the psychological consequences. The present study helps to shed light on the cognitive consequences of the pandemic as it shows that how people remember and imagine negative and positive events is related to their current level of psychological distress. This distress will inevitably fluctuate as the situation with the pandemic continues to change. Thus, it is of the utmost importance to understand how people are remembering and thinking about events in light of the Covid-19 pandemic. This knowledge will aid in the development of at-home therapies and other social-distancing treatments to limit the psychological consequences of the pandemic.

## Footnotes

1 Out of the 904 participants, 284 provided all four descriptions (negative and positive past and future events), 145 provided three out of four descriptions, 114 provided two out of four descriptions, 195 provided one out of four descriptions and 256 did not provide any description. Their ratings in the AMQ subscales emotion, reliving, and rehearsal were still included in the following analyses.

2 Prior literature suggests that participant’s gender and age should relate to their psychological symptoms (Culbertson, 1997; Ditlevsen & Elklit, 2010; Girgus & Yang, 2015; Nolen-Hoeksema & Aldao, 2011). Further, one may expect differences in the psychological symptoms, depression in particular, between participants in the USA and Germany (see Kessler & Bromet, 2013 for review). We therefore tested for effects of gender, age, and country of residence on participants’ depression, anxiety, and stress scores. The results are in the Online supplement. Females reported higher levels of stress and anxiety than men. The older the participants, the lower their stress, anxiety and depression symptoms. Participants residing in the USA reported higher levels of stress and anxiety than those residing in Germany, but, surprisingly, there was no difference in depression between the USA and Germany.

3 One participant did not complete the AMQ, two further participants did not complete the questionnaire after the AMQ for past negative events, two further participants did not complete the questionnaire after the AMQ for future negative events, and two further participants did not complete the questionnaire after the AMQ for past positive events. The linear mixed regression modeling allowed us to include their existing data nonetheless.

4 To check whether our current results were influenced by country of residence, gender, or age of the participants, we ran, in a first step, models that additionally included these predictors. The results that are reported here did not change by inclusion of these additional predictors. We therefore focus on the current models here.
